# Burn-induced Bone Loss: Importance, Mechanisms, and Management

**Published:** 2006-08-08

**Authors:** Gordon L. Klein

**Affiliations:** Shriners Burns Hospital and the University of Texas Medical Branch, Department of Pediatrics, Galveston, TX

## Abstract

**Objective:** The purpose of this article is to familiarize the reader with the issue of bone loss that accompanies severe burn injury. Why is this important? How does it happen? How can we treat it? **Methods:** The published findings on this subject are reviewed and integrated into a conceptual framework. **Results:** Bone loss occurs quickly following a severe burn, is sustained, and increases the risk of postburn fracture. The likely mechanisms responsible are the increase in endogenous glucocorticoid production resulting from the stress response and resorptive cytokines resulting from the systemic inflammatory response and likely aggravated by progressive vitamin D deficiency. Calcium metabolism is also disrupted as the patients develop hypocalcemic hypoparathyroidism likely due to an upregulation of the parathyroid calcium-sensing receptor, possibly due to inflammatory cytokine stimulation. Treatment is achieved by use of anabolic agents and vitamin D supplementation. Studies of acute administration of the antiresorptive agent pamidronate are also promising. **Conclusion:** Postburn bone loss should be looked for in patients with a burn injury of 40% or greater total body surface area. The cause is inherent to the adaptive mechanisms following burn injury. Methods are available to treat this condition.

There is now incontrovertible evidence that bone and calcium metabolism are adversely affected by severe burn injury. In adults, intraoperative iliac crest bone biopsies demonstrated reduced bone formation approximately 3 weeks postburn.^1^ While the effects of these histomorphometric findings on bone density and fracture incidence have not been pursued, similar findings have been found in children with a burn injury of 40% or greater total body surface area (TBSA)^2^ with associated reduction in bone density and an increase in extrapolated annual fracture incidence by nearly twofold in boys and by approximately one third in girls.^3^ There are no similar data available for adults, including firefighters, who have been severely burned, in part because postburn fracture incidence is not tracked by any database. Furthermore, the mechanisms involved in bone loss may be related to specific types of adaptive processes that occur in response to the burn injury, such as the stress and inflammatory responses seen postburn. To the extent that these processes occur in other settings, the findings in burn victims may also apply to other clinical conditions in which bone loss was not suspected.

## SKELETAL CONSEQUENCES OF BURNS

The mechanisms to be covered concern 2 discrete alterations in bone and calcium metabolism following the burn injury: the first is actual bone loss and the second is disordered calcium metabolism. The actual relationship between the 2 effects is not completely clear. After a description of the operative mechanisms to the extent they are known, we will attempt to draw a connection between disordered calcium metabolism and bone loss.

### Bone loss

The bone loss is manifested by an approximate 2% loss of total body bone mineral content (BMC) by 2 months postburn, increasing to about 3.5% by 6 months, with lumbar spine BMC falling by about 8% by 2 months postburn and remaining the same at 6 months postburn,^4^ as determined by dual-energy x-ray absorptiometry (DXA) on a QDR 4500A absorptiometer (Hologics, Waltham, MA). While growth velocity is reduced for the first year postburn^5^ and DXA tends to underread density for smaller children,^6^ the fall in BMC and bone mineral density (BMD) when measured longitudinally remain valid because actual height is not lost. However, because DXA measurements are areal, that is, 2-dimensional rather than 3-dimensional, we cannot be certain how much bone strength is lost if there is a compensatory increase in bone volume. In addition, countering any compensatory increase in bone width to try to maintain strength is the loss of skeletal loading secondary to muscle atrophy resulting from hypermetabolism and negative nitrogen balance.^7^

The way we believe this bone loss occurs is as follows. Within 24 hours of burn injury, there is a rise in proinflammatory cytokines, notably interleukin (IL)-1β and IL-6.^8^ Both of these cytokines stimulate osteoblasts to increase production of the ligand of the receptor activator of nuclear transcription factor κB (RANK ligand, or RANKL), which stimulates marrow stromal cells to differentiate into osteoclasts, resulting in an increase in bone resorption.^9^ Moreover, at some time before 2 weeks postburn, there is a rise in endogenous glucocorticoid production, which, as expressed by measurements of urine free cortisol, can reach an 8-fold elevation above normal.^2^,^10^ Glucocorticoids can acutely stimulate osteoblast production of RANKL,^11^ thus further increasing osteoclastogenesis and bone resorption. This acute surge in bone resorption has not yet been documented but all the causative mechanisms are in place to produce it.

Moving on in time, urine free cortisol excretion remains elevated for the entire period of the acute burn, at least 3 months.^12^ Subsequent effects of glucocorticoid on bone apparent by 2 weeks postburn are an absence of osteoblasts on the bone surface and reduced biochemical markers of marrow stromal cell differentiation into osteoblasts, such as alkaline phosphatase bone morphogenetic protein, and core-binding factor antigen-1 (cbfa-1).^10^ Thus, because osteoblasts are now largely absent, bone resorption cannot be elevated, even in the presence of high circulating levels of IL-1β and IL-6, which are 3-fold and 100-fold elevated, respectively. Urinary deoxypyridinoline, a biomarker of collagen cross-links resulting from resorption, is detectable but the levels are below those for age-related controls.^2^ However, given the reduction in osteoblast differentiation and apoptosis, the likely explanation for the disappearance of osteoblasts, bone formation is virtually absent and bone loss continues. Thus, bone is lost because of the acute effect of endogenous steroids, the presumed synergistic pro-resorptive effects of the endogenous glucocorticoids and cytokines that acutely increase resorption and shut down formation, and the loss of skeletal loading.

### Disordered calcium metabolism

Another early finding, appearing within days after burn injury, is hypocalcemia. This occurs despite the provision of 2.7 g/(m^2^ d) in the diet,^13^ which exceeds the daily reference intake of calcium, and despite frequent parenteral calcium supplementation as needed, determined to be 50 to 70 mg/(kg d).^4^ Furthermore, urinary calcium excretion is elevated. In children this elevation is twice the normal value,^2^,^4^ and in a burned sheep model, fractional excretion of calcium is not different from sham-burned animals despite significantly lower blood calcium.^14^ Moreover, serum concentration of parathyroid hormone (PTH) in children is inappropriately low for the blood calcium levels,^13^ indicating that children with burns have not only hypocalcemia and hypercalciuria but hypoparathyroidism as well. The modified Ellsworth-Howard test, in which PTH is administered subcutaneously and an increase in urinary cyclic AMP and phosphate excretion is observed, produced only a blunted response in children with burns, suggesting PTH resistance.^13^ All these findings could be explained by magnesium depletion, and a good explanation for this hypothesis is that burn patients are resuscitated with Ringer's lactate solution, which does not contain magnesium.^14^

Using the magnesium infusion-retention test described by Rude,^13^,^15^ all children with a burn injury of 40% or greater TBSA at approximately 2 weeks postburn were magnesium depleted.^13^ However, when half of the children were magnesium repleted and PTH and calcium were reexamined, the magnesium-replete children did not have either PTH or calcium levels significantly higher than their magnesium-depleted counterparts.^16^ Thus magnesium depletion, while present, was not the explanation for this disordered calcium metabolism.

### The calcium-sensing receptor

The calcium-sensing receptor, a membrane-bound G-protein-coupled receptor is present primarily but not exclusively on the chief cells of the parathyroid gland. It has the ability to sense both blood calcium and magnesium concentrations.^17^ The exact mechanism of upregulation or downregulation has not been published. With upregulation, as in hypercalciuric hypocalcemia,^17^ the serum PTH levels are inappropriately low. It takes less circulating calcium to reduce parathyroid gland secretion of PTH. The consequence of this phenomenon is a *reduced set point* for calcium stimulation of PTH secretion. This is exactly what we see in children with burns, with low PTH levels, low blood ionized calcium, and high urinary calcium.^13^ Conversely, with primary or secondary hyperparathyroidism, there is a downregulation of the calcium-sensing receptor, indicating that it takes higher than normal concentrations of circulating calcium to turn off the signal for PTH secretion, resulting in hypercalcemia and hyperparathyroidism.

Studies of burned and sham-burned sheep in our laboratory and in collaboration with the laboratory of Brown and colleagues in Boston^14^ have demonstrated a 50% upregulation of the calcium-sensing receptor in the parathyroids of the burned sheep compared to the sham-burned controls, thus providing evidence supporting calcium receptor upregulation as the mechanism that may modify calcium metabolism following burn injury. Furthermore, studies by Nielsen et al in Brown's laboratory using bovine parathyroid chief cells,^18^ Toribio et al^19^ using horse parathyroid, and Canaff and Hendy^20^ demonstrated that both IL-1β^18^,^19^ and IL-6^19^,^20^ are capable of up-regulating the parathyroid gland calcium-sensing receptor with the consequences described above.

### How do bone loss and disordered calcium metabolism interact?

While the upregulation of the calcium-sensing receptor may explain the hypocalcemia, hypercalciuria, and hypoparathyroidism we observe following burn injury, is there a way that these 2 phenomena may interact? It is not that we know for certain all the ways in which these findings play on one another, but at least 2 types of interaction can be postulated. These form a unique adaptation of the body to burn injury. One way in which these 2 adaptations may interact is that the excessive endogenous glucocorticoid production drastically reduces surface osteoblasts available to interact with the proinflammatory cytokines to produce osteoclastogenesis. Thus it is possible that in the absence of osteoblasts, the parathyroid chief cells may be a secondary target for the proinflammatory cytokines, which would then result in the hypocalcemic, hypoparathyroid state observed following burn injury. The second interaction then could be considered to be that the absence of osteoblasts will indirectly result in the hypoparathyroid-associated urinary calcium wasting, making it impossible to deposit calcium in bone.

And the inflammatory response, which results in high levels of pro-inflammatory cytokines, combine to produce the complex of events described above. Of note is that these adaptive responses can be seen in other conditions and should be deemed capable of producing similar effects on bone and calcium metabolism.

### Loose ends: Zinc and vitamin D

To make it clear that we do not have a neat package, we need to acknowledge that burn injuries also create other disturbances in metabolism that may interfere with bone and calcium homeostasis. While there may yet be many unidentified factors that could play a role in the pathogenesis of bone loss, two nutrients that burn patients are known to be deficient in are zinc and vitamin D.

Zinc plays a role in the cross-linking of molecules of type I collagen. It is reported to come out of bone following burn injury.^21^ Furthermore, plasma zinc remains low while urinary zinc excretion is elevated after a burn.^22^ In addition, zinc plays a role in the inflammatory response, and the concentration of zinc in the burn wound exudates is consistently higher than it is in plasma.^22^ Requirements for zinc during recuperation from burn injury have not been established.

More is known about the problem of vitamin D deficiency in burns, although the implications of this finding are still not clear. At Shriners Burns Hospital in Galveston, all patients receive at least 400 IU/d of vitamin D, representing the former recommended daily allowance set by the Food and Nutrition Board of the Institute of Medicine of the National Academy of Sciences. It represents twice the daily reference intake that this same institution has used to replace the recommended dietary allowances. Thus 200 IU/d of vitamin D is now the established recommendation for children.^23^ Vitamin D deficiency by definition is the lower-than-normal serum concentration of 25-hydroxy-vitamin D (25(OH)D), the main circulating form of vitamin D after undergoing 25-hydroxylation in the liver by the cytochrome p450–dependent enzyme vitamin D 25-hydroxylase. There is, however, debate over whether the lower limit of circulating 25(OH)D should be reset to one in which serum PTH levels are normal, currently recommended to be 30 ng/mL, a 33% increase in the lower limit of normal levels.

It is not known exactly when vitamin D deficiency develops following a burn injury. While serum vitamin D levels are lower by 3 weeks following burn injury, it is not possible to evaluate the status of 25(OH)D because free 25(OH)D is no longer measured nor are there definitions of vitamin D sufficiency or deficiency based on free 25(OH)D. However, by 6 months postburn, most constitutive proteins are back to normal.^24^ As early as 14 months after burn injury, serum 25(OH)D levels are low.^25^ A previous cross-sectional study looking at children at 2 and at 7 years postburn^26^ showed that at 2 years, nearly all serum levels of 25(OH)D were low, while levels of the biologically active metabolite 1α,25-di-hydroxyvitamin D (1,25(OH)_2_D) were all normal. However, at 7 years postburn, not only were all the serum levels of 25(OH)D low but half of the circulating levels of 1,25(OH)_2_D were also low, suggesting a progressive deficiency.

Why is vitamin D deficiency important in burns? We have evidence of a direct relationship between serum levels of 25(OH)D and lumbar spine bone mineral density *Z* score.^26^ Furthermore, correlations between serum levels of 25(OH)D and bone density have been shown in other studies.^27^–^30^ Postdischarge supplementation with a multivitamin containing 400 IU has been recommended^25^,^26^ because following a burn injury, the skin becomes biochemically abnormal. Burn scar and adjacent normal-appearing skin not only have their capacity to convert 7-dehydrocholesterol, the vitamin D precursor in skin, to pre-vitamin D_3_ reduced to 25–30% of normal, but they will also contain reduced amounts of the 7-dehydrocholesterol substrate.^25^ This finding may indicate a persistent biochemical abnormality of skin cholesterol biosynthesis of indefinite duration following a burn.

## MANAGEMENT

Published studies of the anabolic agents recombinant human growth hormone^31^ and oxandrolone^32^ have shown that there is an increase in lean body mass after 6 months of daily use, and only at 1 year postburn is there a significant increase in BMC as seen by DXA studies. Furthermore, with neither drug is there an increase in areal BMD of the lumbar spine, only an increase in BMC of the total body and lumbar spine. Because areal BMD is the quotient of BMC and bone area, the increase in BMC and not BMD implies a proportionate increase in bone area in patients treated with these anabolic agents. Thus the bone becomes bigger and biomechanically stronger and theoretically less likely to fracture. However, because BMD *Z* scores remain below normal^31^,^32^ the amount of mineralization per unit area (BMD) of the bone does not differ between treated and untreated subjects. Whether the decreased BMD still constitutes a risk factor for fractures is unknown. Therefore, the trial of an antiresorptive agent such as a bisphosphonate given acutely after burn injury might result in the preservation of BMD.

Preliminary data published in 2005 indicate that intravenous administration of the bisphosphonate pamidronate at a dose of 1.5 mg/kg in 5% dextrose and water over 12 hours within the first 10 days of burn injury and then again 1 week later to children with a burn injury of 40% or greater TBSA resulted in prevention of bone loss for the first 6 months postburn, with trabecular, or cancellous, bone more greatly affected than cortical bone and without any increase in postburn hypocalcemia.^4^

Thus while we still do not fully understand all the effects of burn injury on bone metabolism, the use of antiresorptives, anabolic agents, and vitamin D should minimize the damage to bone and calcium metabolism resulting from the burn injury. Furthermore, the understanding obtained from the study of burn effects on bone and calcium metabolism may result in beneficial applications to the study and treatment of other conditions in which similar adaptive responses are operative.
